# Supplementary material for: INPP5K and Atlastin-1 maintain the nonuniform distribution of ER–plasma membrane contacts in neurons
Source: Life Sci Alliance. 2021 Sep 23;4(11):e202101092. doi: 10.26508/lsa.202101092 (PMC8507493; doi:10.26508/lsa.202101092)
Supplement: Supplementary file 14 [file LSA-2021-01092_TableS1.docx]

**Supplementary Table 1**

**List of primers**

| **Oligonucleotides** | **Source** | **Identifier** |
| --- | --- | --- |
| AAAAAATGCTCGAGACGGTACCGGTAGAAAAAATGTCCAAGGGAGAGGAGCTTTTCACCG | This Paper | splitGFP1-10_Xhol_F |
| CTCAGTTGGAATTCTCAGGATCCTCCTCCTCCGGTTCCCTTCTCGTTTGGGTCCTTGGAA | This Paper | splitGFP1-10_EcoRl_R |
| GACCCTTGGCTAGCATGCGAGATCATATGGTTCTGCACGA | This Paper | splitGFP11_3xPH-PLCδ1_Nhel_F |
| TTTCTACCGGTACCTGCTTCTGTCTTTGATCCATTGAACC | This Paper | splitGFP11_3xPH-PLCδ1_Kpnl_R |
| CTCAGTTGGAATTCTTAGTGCTTCTGTCTTTGATCCATTG | This Paper | splitGFP11_3xPH-PLCδ1 _EcoRl_R |
| ACGAATTGGGCGCGCCATGGTCAGCAAGGGAGAGGCAGTTATCAA | This Paper | wrmScarlet_Ascl_F |
| ATGTCGACGAATTCGCTAGCCTTGTAGAGCTCGTCCATTCCTCCG | This Paper | wrmScarlet_EcoRl_R |
| GGTGGCAAATTCATGACGGTACCGGTAGAAAAAATGCTCGAG | This Paper | splitGFP11_mutagenesis_F |
| TGTAATGCCGGCAGCGTTGACGTATTCGTGCAGAACCATATG | This Paper | splitGFP11_mutagenesis_R |
| CCAGAGCTCACCTAGGATCTATTCCAGAGTTCGTTCCCGA | This Paper | pMLS262_itr-1pB_Avrll_F |
| CAAACTTGGGCCGGCCCAATTCGTGTGCTTCCACCACCAC | This Paper | pMLS262_itr-1pB_Fsel_R |
| AGAGCTCACCTAGGATCTCAACATAGTAGATTTTTAAAAA | This Paper | pMLS262_odr-3p_Avrll_F |
| CAAACTTGGGCCGGCCATCTAAAAAAACAATGATCTATGA | This Paper | pMLS262_odr-3p_Fsel_R |
| GACCCTTGGCTAGCATGGATTGGAAAATAACAATATTCACATACA | This Paper | cil-1_Nhel_F |
| TTTCTACCGGTACCGTCTATGGTTGTTCAACTATTTGAAAGACTT | This Paper | cil-1_stop_Kpnl_R |
| TGGTGACTTCGCCTTTCGCGTAGAAGAGGATGTGAATACA | This Paper | cil-1_N175A_mutagenesis_F |
| AACCAGAATGCTGCTCTGACACTCTTATCCTCTGGAAACG | This Paper | cil-1_N175A_mutagenesis_R |
| GAGACAGCGGTACCGTCTATGCAGCGGGTGCAGTAACTCTAAACA | This Paper | cil-1_ΔSKICH_Kpn1_R |
| AGTGGTACCGATTGGAAAATAACAATATTCACATACAATCTGGCC | This Paper | KpnI-cil-1_S |
| GGTGGATCCCTATGCAGCGGGTGCAGTAACTCTAAACATTG | This Paper | BamHI-stop-cil-1_AS |
| TAGAACATTTTCAGGAGGACCCTTGGCTAGCATGGGCTGCGTGTGCAGCAGCAACCCCGA | This Paper | Lck_mScarlet_5ptase_CIL-1_F |
| TGGTAGCGACCGGCGCTCAGTTGGAATTCCTATGCAGCGGGTGCAGTAACTCTAAACATT | This Paper | Lck_mScarlet_5ptase_CIL-1_R |
| ACGAATTGGGCGCGCCATGATTTTACTTATTCTCACTTCGATATT | This Paper | CP450_Ascl_F |
| CAATCCATGCTAGCATATCTTTCCTTCATTGAAACTCTGTAATTC | This Paper | CP450_Nhel_R |
| ACGAATTGGGCGCGCCATGAAATTCTCACTCTTCGCCCTA | This Paper | TRAPbeta_T04G9.5_AscI_F |
| GGTCCTCCCCCGGGACAGTAGACTTCTTCTTGATAACATT | This Paper | TRAPbeta_T04G9.5_SmaI_R |
| ACTGACTGGGCCGGCCCTGACACTAAGTTTCTCTGCAGTGTTACACGAAC | This Paper | mig-13p_Fsel_F |
| CCTCTAGAGGCGCGCCTACCTGAAATTCTGAATTAAATGATAATTGAAGA | This Paper | mig-13p_Ascl_R |
| ACGAATTGGGCGCGCCATGGAAACAACTCCTCAAAACGAG | This Paper | atln-1_AscI_F |
| AATACCATGGTACCCTAATGCCGTTTTCTGAGTCCATCAG | This Paper | atln-1_stop_KpnI_R |
| GGAGCTTCTCAAGTACTTTAAGGCTTATATGCACATTTTCAGAGGCCAAG | This Paper | atln-1_E338K_site mutation_F |
| CGGCACGTCATCTTTTGACCGTTAATCTCCTTATGAACGAGAGCGTGTGA | This Paper | atln-1_E338K_site mutation_R |
| TCGTAAAGGAGCATCCTTCCTTTTAAACTTTTTCCTTCGTTATTTGACAT | This Paper | atln-1_K80A_site mutation_F |
| TATGCACCGGCAACTCCGATTACGGCAACCTTTTTATCGGCGACTTTCGG | This Paper | atln-1_K80A_site mutation_R |
| AAAGATATGCTAGCCAAGATGTCAAAATTTGTGTTTCAACATTTA | This Paper | ocrl-1_IPPc_Nhel-F |
| CTCACCATCTCGAGCTAATTGATTTTCTTTACTTTCAAATTGAAC | This Paper | ocrl-1_IPPc_XhoI-R |
| AAAGATATGCTAGCCAATCCATTAAAATATTCGTTGGAACTTGGA | This Paper | unc-26_IPPc_Nhel-F |
| GAGACAGCGGTACCGTCTAGCCAACTTTAAAAGTTTCCACTTTGA | This Paper | unc-26_IPPc_Kpnl-R |
| TGGTAGCGACCGGCGCTCAGTTGGAATTCCTAATTGATTTTCTTTACTTTCAAATTGAAC | This Paper | Lck_mScarlet_5ptase_OCRL-1_R |
| ATGGTAGCGACCGGCGCTCAGTTGGAATTCCTAGCCAACTTTAAAAGTTTCCACTTTGAA | This Paper | Lck_mScarlet_5ptase_UNC-26_R |
| TGTACAAGGCTAGCATGCCCTCGGCCAAACAAAGGGGCTCCAAGG | This Paper | CLIMP-63_NheI-F |
| AATACCATGGTACCTTAGACCTTTTCGTGAATCTTCTCCACTTTC | This Paper | CLIMP-63_KpnI-R |
| AGATCTCGAGGCCACCATGATTTTACTTATTCTCACTTCGATATT | This Paper | XhoI-CP450_S |
| CCGGGCCCCATTGATTTTCTTTACTTTCAAATTGAACATGGCTCG | This Paper | ApaI-ocrl1_AS |
| CCCGGGCCCCGCCAACTTTAAAAGTTTCCACTTTGAAAACAGC | This Paper | ApaI-unc-26_AS |
| ggcTGTACACAAGATGTCAAAATTTGTGTTTCAACATTTAATGTG | This Paper | BsrGI-ocrl1_S |
| GGTGGATCCCTAATTGATTTTCTTTACTTTCAAATTGAACATGGC | This Paper | BamHI-stop-ocrl1 _AS |
| AGTGGTACCCAATCCATTAAAATATTCGTTGGAACTTGGAATGTG | This Paper | KpnI_unc-26_S |
| GGTaGATCTCTAGCCAACTTTAAAAGTTTCCACTTTGAAAACAG | This Paper | BglII-stop-unc-26_AS |

**List of plasmids**

| **Plasmid** | **Source** | **Identifier** |
| --- | --- | --- |
| *itr-1pB::CP450::splitGFP1-10* | This Paper | JB_96 |
| *itr-1pB::splitGFP11::3xPH^PLCδ1^*::mCherry | This Paper | JB_68 |
| *itr-1pB::splitGFP11::3xPH^PLCδ1^* | This Paper | JB_124 |
| *itr-1pB::mNeonGreen::ESYT-2* | This Paper | JB_73 |
| *itr-1pB::wrmScarlet::ESYT-2* | This Paper | JB_123 |
| *itr-1pB::split GFP11::mCherry* | This Paper | JB_67 |
| *itr-1pB::FLP* | This Paper | JB_29 |
| *odr-3p::FLP* | This Paper | RAI_4 |
| *itr-1pB::cil-1* | This Paper | JB_107 |
| *itr-1pB::cil-1^N175A^* | This Paper | JB_120 |
| *itr-1pB::cil-1^ΔSKICH^* | This Paper | JB_117 |
| *itr-1pB::cil-1^PM^* | This Paper | JB_185 |
| *itr-1pB::cil-1^ER^* | This Paper | JB_126 |
| *itr-1pB::TRAPbeta::mNeonGreen* | This Paper | RAI_28 |
| *mig-13p::splitGFP11::mCherry* | This Paper | JB_137 |
| *itr-1pB::atln-1* | This Paper | RAI_9 |
| *itr-1pB::atln-1^E338K^* | This Paper | RAI_14 |
| *itr-1pB::atln-1^K80A^* | This Paper | RAI_21 |
| *itr-1pB::CP450::mCherry* | This Paper | JB_86 |
| *itr-1pB::ocrl-1^ER^* | This Paper | JB_150 |
| *itr-1pB::unc-26^ER^* | This Paper | JB_144 |
| *itr-1pB::ocrl-1^PM^* | This Paper | JB_186 |
| *itr-1pB::unc-26^PM^* | This Paper | JB_187 |
| *itr-1pB::mCherry::CLIMP-63* | This Paper | JB_174 |
| *OCRL-1^ER^-EGFP* | This Paper | TN300 |
| *UNC-26^ER^-EGFP* | This Paper | TN301 |
| *mScarlet-I-CIL-1^PM^* | This Paper | TN305 |
| *mScarlet-I-OCRL-1^PM^* | This Paper | TN306 |
| *mScarlet-I-UNC-26^PM^* | This Paper | TN307 |
| *Lck-mScarlet-I* | Addgene | #98821 |
| *CLIMP-63 pcDNA* | Addgene | #80977 |
| *itr-1pB::cb5::mCherry* | This Paper | JB87 |
| *iRFP-PH^PLCδ1^* | De Camili lab (Yale University) |  |
