# Supplementary material for: INPP5K and Atlastin-1 maintain the nonuniform distribution of ER–plasma membrane contacts in neurons
Source: Life Sci Alliance. 2021 Sep 23;4(11):e202101092. doi: 10.26508/lsa.202101092 (PMC8507493; doi:10.26508/lsa.202101092)
Supplement: Supplementary file 15 [file LSA-2021-01092_TableS2.docx]

**Supplementary Table 2**

**List of strains**

SAH identifies *C. elegans* strains that were generated for the current study.

| **Strain** | **Genotype** | **Source** | **Figure** |
| --- | --- | --- | --- |
| N2 | *Wild-type Bristol strain* | Caenorhabditis Genetics Center (CGC) | 1A, S3A, S3D, S3E |
| SAH553 | *sybIs50 X [itr-1pB::mNeonGreen::esyt-2 (0.5ng/μl); itr-1pB::mCherry::rab-3 (5ng/μl); pCFJ90 - myo-2p::mCherry (1ng/μl)]* | This paper | 3A, 3C, 3F, 3G, 4C, 4E-4G, 5E-5I, 6A, 6C, S3F, S4C, S5G, S5H, S6B-S6G, S7C, S7D |
| SAH526 | *sybIs2669 X [itr-1pB::CP450::splitGFP1-10 (10ng/μl); itr-1pB::splitGFP11::mCherry (10ng/μl); pCFJ90 - myo-2p::mCherry (2ng/μl)]* | This paper | 4I, 5D, 6E |
| XE1931 | *wpIs101 [itr-1pB::GFP::rab-3::SL2::mCherry; myo-2p::mCherry]; ric-7(n2657) V* | The Hammarlund Lab | 7D-7G |
| SAH91 | *esyt-2 (yas27 [EGFP^FLP-on::esyt-2]) III; unc-119(ed3) III; yasEx32 [itr-1pB::FLP (10ng/μl); itr-1pB::mCherry::rab-3 (10ng/μl)]* | This paper | 2A-2D, S2A-S2C |
| SAH289 | *yasEX111 [itr-1pB:CP450::splitGFP1-10 (1ng/μl); itr-1pB::splitGFP11::3xPH-PLCδ1::mCherry (1ng/μl); pCFJ9­0 - myo-2p::mCherry (2ng/μl)]* | This paper | 1D-1G |
| SAH318 | *atln-1(yas38) IV; sybIs50 X* | This paper | 5A, 5B, 5E-5I, S3C-S3F, S5G, S5H |
| SAH319 | *esyt-2 (yas27 [EGFP^FLP-on::esyt-2]) III; unc-119(ed3) III; yasEx112 [odr-3p::FLP (10ng/μl); odr-3p::mCherry::rab-3 (10ng/μl)]* | This paper | S2E |
| SAH340 | *cil-1(yas37)/qC1 [dpy-19(e1259) glp-1(q339)] nIs189 III; sybIs50 X* | This paper | 3B, 3D, 3F, 3G, 4C, 4E-4G, 5E-5I, S3B, S3D, S3F, S4C |
| SAH368 | *cil-1(yas37)/qC1 [dpy-19(e1259) glp-1(q339)] nIs189 III; sybIs50 X; yasEx142 [itr-1pB::cil-1 (5ng/μl); pCFJ104 - myo-3p::mCherry (5ng/μl)]* | This paper | 3E-3G |
| SAH386 | *cil-1(yas37)/qC1 [dpy-19(e1259) glp-1(q339)] nIs189 III; ric-7(n2657) V; wpIs101* | This paper | 7F-7G |
| SAH387 | *atln-1(yas38)/tmC25 [unc-5(tmIs1241)] IV; sybIs50 X; yasEx117 [itr-1pB::atln-1 (5ng/μl); elt-2p::mCherry (10ng/μl)]* | This paper | S5C, S5D, S5G, S5H |
| SAH388 | *cil-1(yas37)/qC1 [dpy-19(e1259) glp-1(q339)] nIs189 III; sybIs50 X; yasEx118 [itr-1pB::atln-1 (5ng/ul); elt-2p::mCherry (10ng/μl)]* | This paper | 5E-5I |
| SAH397 | *cil-1(yas37)/qC1 [dpy-19(e1259) glp-1(q339)] nIs189 III; sybIs50 X; yasEx158 [itr-1pB::mCherry::cil-1 N175A::SL2::mCherry (5ng/μl); elt-2p::mCherry (10ng/μl)]* | This paper | 4C, S4A, S4C |
| SAH398 | *cil-1(my15) III; sybIs50 X* | This paper | 4C, S4B, S4C |
| SAH405 | *atln-1(yas38)/tmC25 [unc-5(tmIs1241)] IV; ric-7(n2657) V; wpIs101* | This paper | 7F-7G |
| SAH407 | *atln-1(yas38)/tmC25 [unc-5(tmIs1241)] IV; ric-7(n2657) V; wpIs101; yasEx121 [itr-1pB::atln-1 (5ng/μl); ofm-1p::GFP (10ng/μl)]* | This paper | 7F-7G |
| SAH411 | *esyt-2 (syb709) III;* *ric-7(n2657) V; wpIs101* | This paper | 7D-7E |
| SAH412 | *ret-1(tm390) V; sybIs50 X* | This paper | 6A-6C, S6A-S6B |
| SAH429 | *atln-1(yas38)/tmC25 [unc-5(tmIs1241)] IV; sybIs50 X; yasEx124 [itr-1pB:: atln-1 E338K (5ng/μl); elt-2p::mCherry (10ng/μl)]* | This paper | S5C, S5D, S5G, S5H |
| SAH439 | *cil-1(yas37)/qC1 [dpy-19(e1259) glp-1(q339)] nIs189 III; ric-7(n2657) V; wpIs101; yasEX128 [itr-1pB::cil-1 (5ng/μl); ofm-1p::GFP (10ng/μl)]* | This paper | 7F-7G |
| SAH445 | *cil-1(yas37)/qC1 [dpy-19(e1259) glp-1(q339)] nIs189 III; sybIs50 X; yasEx134 [itr-1pB::cil-1* *ΔSKICH (5ng/μl); elt-2p::mCherry (10ng/μl)]* | This paper | 4C, S4A, S4C |
| SAH446 | *atln-1(yas38)/tmC25 [unc-5(tmIs1241)] IV; sybIs50 X; yasEx135 [itr-1pB::cil-1 (5ng/μl); elt-2p::mCherry (10ng/μl)]* | This paper | 5E-5G |
| SAH450 | *cil-1(yas37)/qC1 [dpy-19(e1259) glp-1(q339)] nIs189 III; sybIs50 X; yasEx139 [itr-1pB::cil-1 (ER) (5ng/μl); elt-2p::mCherry (10ng/μl)]* | This paper | 4B, 4C, S4C |
| SAH482 | *cil-1(yas37)/qC1 [dpy-19(e1259) glp-1(q339)] nIs189 III; atln-1(yas38) IV; sybIs50 X* | This paper | 5E-5G |
| SAH483 | *atln-1(yas38)/tmC25 [unc-5(tmIs1241)] IV; sybIs50 X; yasEx161 [itr-1pB::atln-1 K80A (5ng/μl); elt-2p::mCherry (10ng/μl)]* | This paper | S5C, S5D, S5G, S5H |
| SAH484 | *sybIs50 X; yasEx162 [itr-1pB:: atln-1 K80A (5ng/μl); elt-2p::mCherry (10ng/μl)]* | This paper | S5E-S5H |
| SAH486 | *cil-1(yas37)/ qC1 [dpy-19(e1259) glp-1(q339)] nIs189 III; ric-7(n2657) V; wpIs101; yasEX164 [itr-1pB::atln-1 (5ng/μl); ofm-1p::GFP (10ng/μl)]* | This paper | 7F-7G |
| SAH488 | *yasEx166 [itr-1pB::CP450::splitGFP1-10 (10ng/μl); itr-1pB::splitGFP11::mCherry (10ng/μl); pCFJ90 - myo-2p::mCherry (2ng/μl)]* | This paper | S1A-S1D |
| SAH489 | *yasEx167 [itr-1pB::wrmScarlet::esyt-2 (3ng/μl); itr-1pB::CP450::splitGFP1-10 (3ng/μl); splitGFP11::3xPH-PLCδ1 (3ng/μl); pCFJ90 - myo-2p::mCherry (2 ng/μl)]* | This paper | 2E, 2F |
| SAH492 | *sec-61.B (knu560 [Nterminal splitGFP1-10]) IV; yasEx170 [mig-13p::splitGFP11::mCherry (10ng/μl); elt-2p::mCherry (10ng/μl)]* | This paper | 6F-6H, S6H |
| SAH493 | *sec-61.B (knu560 [Nterminal splitGFP1-10]) IV; ret-1(tm390) V; yasEx171 [mig-13p::splitGFP11::mCherry (10ng/μl); elt-2p::mCherry (10ng/μl)]* | This paper | 6F-6H, S6H |
| SAH494 | *yasEx172 [itr-1pB::cb5::mCherry (5ng/μl); itr-1pB::TRAPbeta::mNeonGreen (4ng/μl); pCFJ90 - myo-2p::mCherry (2ng/μl)]* | This paper | 6I-6K, S6I |
| SAH507 | *yasEx179 [itr-1pB::CP450::mCherry (10ng/μl); pCFJ90 - myo-2p::mCherry (2ng/μl)]* | This paper | S5A |
| SAH528 | *sybIs2669 X; ret-1(tm390) V* | This paper | 6D, 6E |
| SAH530 | *yasEx172 [itr-1pB::cb5::mCherry (5ng/μl); itr-1pB::TRAPbeta::mNeonGreen (4ng/μl); pCFJ90 - myo-2p::mCherry (2ng/μl)]; ret-1(tm390) V* | This paper | 6I-6K, S6I |
| SAH532 | *yasEx172 [itr-1pB::cb5::mCherry (5ng/μl); itr-1pB::TRAPbeta::mNeonGreen (4ng/μl); pCFJ90 - myo-2p::mCherry (2ng/μl)]; cil-1(yas37) qC1 [dpy-19(e1259) glp-1(q339)] nIs189 III* | This paper | 6I-6K, S6I |
| SAH538 | *yasEx172 [itr-1pB::cb5::mCherry (5ng/μl); itr-1pB::TRAPbeta::mNeonGreen (4ng/μl); pCFJ90 - myo-2p::mCherry (2ng/μl)]; atln-1(yas38) IV* | This paper | 6I-6K, S6I |
| SAH543 | *yasEx179 [itr-1pB::CP450::mCherry (10ng/μl); pCFJ90 - myo-2p::mCherry (2ng/μl)]; atln-1(yas38)/tmC25 [unc-5(tmIs1241)] IV* | This paper | S5A |
| SAH547 | *yasEx179 [itr-1pB::CP450::mCherry (10ng/μl); pCFJ90 - myo-2p::mCherry (2ng/μl)]; cil-1(yas37)/ qC1 [dpy-19(e1259) glp-1(q339)] nIs189 III* | This paper | S5A |
| SAH549 | *sybIs2669 X; cil-1(yas37)/ qC1 [dpy-19(e1259) glp-1(q339)] nIs189 III* | This paper | 4H, 4I |
| SAH550 | *sybIs2669 X; atln-1(yas38)/tmC25 [unc-5(tmIs1241)] IV* | This paper | 5C, 5D |
| SAH551 | *jph-1(ok2823) I; ric-7(n2657) V; wpIs101* | This paper | 7D, 7E |
| SAH571 | *cil-1(yas37)/qC1 [dpy-19(e1259) glp-1(q339)] nIs189 III; sybIs50 X; yasEx188 [itr-1pB::CP450::unc-26 (ER) (5ng/μl); elt-2p::mCherry (10ng/μl)]* | This paper | 4D, 4E |
| SAH579 | *cil-1(yas37)/qC1 [dpy-19(e1259) glp-1(q339)] nIs189 III; sybIs50 X; yasEx191 [itr-1pB::CP450::ocrl-1 (ER) (5ng/μl); elt-2p::mCherry (10ng/μl)]* | This paper | 4D, 4E |
| SAH562 | *sec-61.B (knu560 [Nterminal splitGFP1-10]) IV;cil-1 (yas37) qC1 [dpy-19(e1259) glp-1(q339)] nIs189 III; yasEx170 [mig-13p::splitGFP11::mCherry (10ng/uL); elt-2p::mCherry (10ng/ul)]* | This paper | 6F-6H, S6H |
| SAH656 | *sybIs2669 X; ric-7(n2657) V* | This paper | S7A, S7B |
| SAH666 | *sybIs50 X; ric-7 (n2657) V; yasEx241 [itr-1pB::mCherry (50ng/ul); pCFJ421 - myo-2p::GFP::H2B (10ng/ul)]* | This paper | S7C, S7D |
| SAH667 | *yasEx232 [itr-1pB::mCherry::CLIMP63 (20ng/ul); pCFJ421 - Pmyo-2::GFP::H2B (5ng/ul)]; sybIs50 X* | This paper | S6C-S6G |
| SAH675 | *cil-1(yas37)/qC1 [dpy-19(e1259) glp-1(q339)] nIs189 III; sybIs50 X; yasEx238 [itr-1pB::cil-1 (PM) (5ng/μl); elt-2p::mCherry (10ng/μl)]* | This paper | 4B, 4C, S4C |
| SAH676 | *cil-1(yas37)/qC1 [dpy-19(e1259) glp-1(q339)] nIs189 III; sybIs50 X; yasEx239 [itr-1pB::unc-26 (PM) (5ng/μl); elt-2p::mCherry (10ng/μl)]* | This paper | 4E |
| SAH677 | *cil-1(yas37)/qC1 [dpy-19(e1259) glp-1(q339)] nIs189 III; sybIs50 X; yasEx240 [itr-1pB::ocrl-1 (PM) (5ng/μl); elt-2p::mCherry (10ng/μl)]* | This paper | 4E |
